# Main group metal-mediated strategies for C–H and C–F bond activation and functionalisation of fluoroarenes

**DOI:** 10.1039/d3sc03548d

**Published:** 2023-09-13

**Authors:** Neil R. Judge, Alessandra Logallo, Eva Hevia

**Affiliations:** a Departement für Chemie, Biochemie und Pharmazie, Universität Bern Switzerland eva.hevia@unibe.ch

## Abstract

With fluoroaromatic compounds increasingly employed as scaffolds in agrochemicals and active pharmaceutical ingredients, the development of methods which facilitate regioselective functionalisation of their C–H and C–F bonds is a frontier of modern synthesis. Along with classical lithiation and nucleophilic aromatic substitution protocols, the vast majority of research efforts have focused on transition metal-mediated transformations enabled by the redox versatilities of these systems. Breaking new ground in this area, recent advances in main group metal chemistry have delineated unique ways in which s-block, Al, Ga and Zn metal complexes can activate this important type of fluorinated molecule. Underpinned by chemical cooperativity, these advances include either the use of heterobimetallic complexes where the combined effect of two metals within a single ligand set enables regioselective low polarity C–H metalation; or the use of novel low valent main group metal complexes supported by special stabilising ligands to induce C–F bond activations. Merging these two different approaches, this Perspective provides an overview of the emerging concept of main-group metal mediated C–H/C–F functionalisation of fluoroarenes. Showcasing the untapped potential that these systems can offer in these processes; focus is placed on how special chemical cooperation is established and how the trapping of key reaction intermediates can inform mechanistic understanding.

## Abstract 2

This *Perspective* showcases recent advances on the use of specially designed main-group metal complexes to facilitate chemoselective C–H/C–F functionalisation of fluoroarenes.

## Graphical abstract

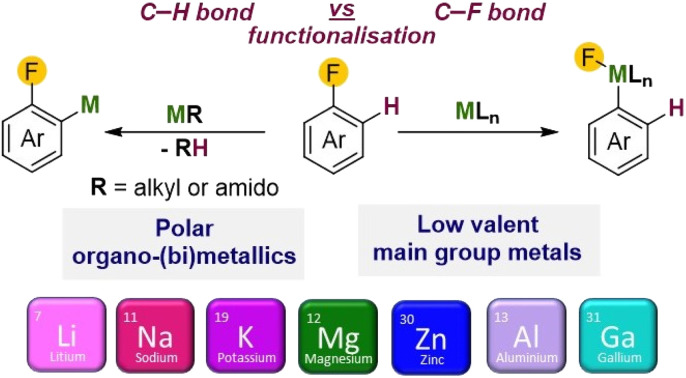

## Introduction

Fluoroarenes are recurrent structural building blocks resident in a variety of agrochemicals and active pharmaceutical ingredients.^1^ The special properties of fluorine can provide organic compounds with enhanced metabolic stability, bioavailability, lipophilicity and binding affinity among other features.^2^ Despite their synthetic relevance and the fact that fluorine is the most abundant halogen in the Earth's crust, fluoroarenes are rarely found in nature so are often synthetically produced.^3^ The synthesis of fluoroarenes usually involves the highly toxic and corrosive gas hydrogen fluoride (HF), however Aldridge and Gouverneur have recently developed a new method to access fluorochemicals *via* a mechanochemical process involving acid grade fluorspar (>97% CaF_2_) and dipotassium hydrogen phosphate (K_2_HPO_4_) by-passing the formation of HF offering a safer approach to the synthesis of these important compounds.^4^ The development of methods for the regioselective functionalisation of C–H and C–F bonds in these molecules is highly sought for in synthesis.

Classical approaches include the use of s-block organometallics for deprotonative metalation or nucleophilic aromatic substitutions of fluoroarenes.^5^ However the highly polar nature of these reagents and the fragility of the relevant metalated intermediates imposes important drawbacks, including limited functional group tolerance and selectivity, requiring in many cases the imposition of strict reaction conditions.^5^ Thus, lithiation of fluoroarenes poses important safety concerns for example, pentafluorophenyllithium (LiC_6_F_5_) is a thermally unstable compound above −25 °C which can explode on warming from cryogenic temperatures.^5*f*^

**Scheme 1:**
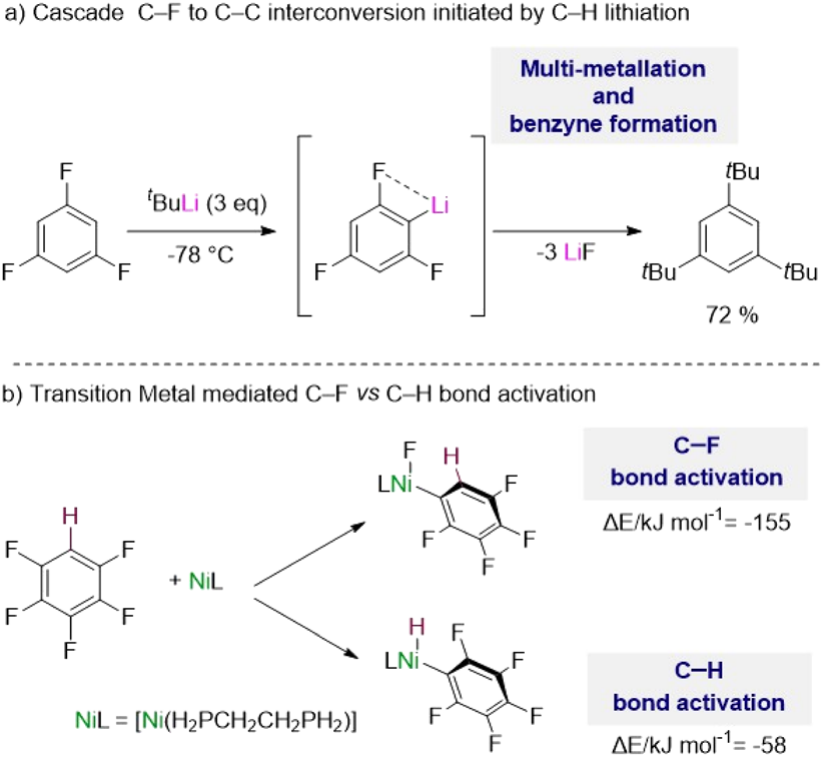
(a) Challenges in the metalation of fluoroarenes using organolithium reagents. (b) Contrasting energies in C–F *vs.* C–H bond activation using transition metals.

The complexity of these reactions is illustrated by Schlosser's seminal study on the lithiation of 1,3,5-trifluorobenzene,^5*b*^ where the initial metalation of this substrate by *t*BuLi at −78 °C triggers multiple hydrogen/lithium interconversions along with benzyne formation (Scheme 1a).

Addressing some of these limitations, transition metal complexes have shown excellent capabilities to activate C–H bonds in fluoroarenes, although in many cases activation of their C–F bonds can compete or being favoured.^1*c*,*d*,6^ C–F bonds are typically described as the strongest bond in organic chemistry, which is attributed to the large electronegativity of fluorine, inducing a large C^*δ*+^–F^*δ*−^ polarization of these bonds.^7^

Thus, while C–H bonds are weaker than C–F bonds, C–F bond activation by the metal complexes is usually more exothermic with the formation of a new M–F bond which provides a significant thermodynamic driving force for the reaction.^1*c*,6^ For example, with the aid of DFT calculations McGrady and Perutz have shown that pentafluorobenzene reacts preferentially with a d^10^ Ni(0) complex [Ni(H_2_PCH_2_CH_2_PH_2_)] *via* oxidative addition of one of its C–F bonds *vs.* the C–H bond by about 100 kJ mol^−1^ (Scheme 1b).^8^

For fluoroarenes the C–F bond energies weaken systematically with the number of *ortho*-fluorine substituents, while the energies of the C–H bonds strengthen.^1*c*^ These changes can have a profound impact on the chemo- and regioselectivity of the reactions, depending on the substrate and the type of metal complex employed.^1*c*,8^

The so called “*ortho* fluorine effect” is a significant factor in the perceived selectivity of the C–H oxidative addition of fluoroarenes occurring preferentially *ortho* to the fluorine atom.^9^ Perutz and Eisenstein have elegantly demonstrated with the aid of experimental and computational studies the thermodynamic preference of C–H bond activation *ortho* to a fluorine substituent.^9*b*^ Investigating a range of transition metal complexes, a correlation between the M–C and C–H bonds was established which revealed a large increase in the M–C bond energy with *ortho*-fluorine substitution on the aryl ring. It has also noted that in cases where a mixture of kinetic and thermodynamic products is observed, the reversible nature of C–H activation of fluoroarenes allows for exclusive formation of the thermodynamic product (over time or upon heating) forming the isomer with the greatest number of *ortho* fluorine substituents to the newly formed M–C bond.^9*a*,*c*^ The increasing fluorine content of the arene increases the strength of the subsequent M–C bond.^9^

Towards more sustainable protocols it should be noted that earth abundant transition metals such as Fe, Co or Ni have already shown good potential to mediate these transformations, typically centred on their ability to change oxidation state, not only stoichiometrically but also under catalytic regimes.^1*c*,10^

In parallel to these studies, recent advances in main group metal chemistry have demonstrated that by combining alkali-metal metals with less electropositive metals such as Mg, Zn or Al within the same molecular scaffold, it is possible to design highly effective and regioselective heterobimetallic bases for arene metalation.^11^ Coined by Mulvey as “alkali-metal mediated metalations” these methods allow for trapping of highly sensitive anions while operating at room temperature.^12^

In addition, in the past decade the field of low valent main group metal complexes has continued to grow in developing applications in small molecule activation.^13^ Supported by specially designed ligands, these metal complexes mostly react as potent nucleophiles^14^ or reducing agents^15^ with a concomitant change in the oxidation state of the main group element.

Though two distinct approaches but having the same aim, to promote C–H/C–F functionalisation of fluoroarenes, it is convenient to combine them in this single *Perspective* on applications of main group metal complexes. Showcasing recent examples where key reaction intermediates have been trapped and structurally defined, the *Perspective* also collects mechanistic proposals on the operation of these emerging main group metal strategies. It must be noted that this *Perspective* focuses on recent advances in the field using s-block metals along with Zn, Ga and Al complexes.^16^

## Regioselective C–H metalation of fluoroarenes: new developments in maingroup metal bases

**Scheme 2:**
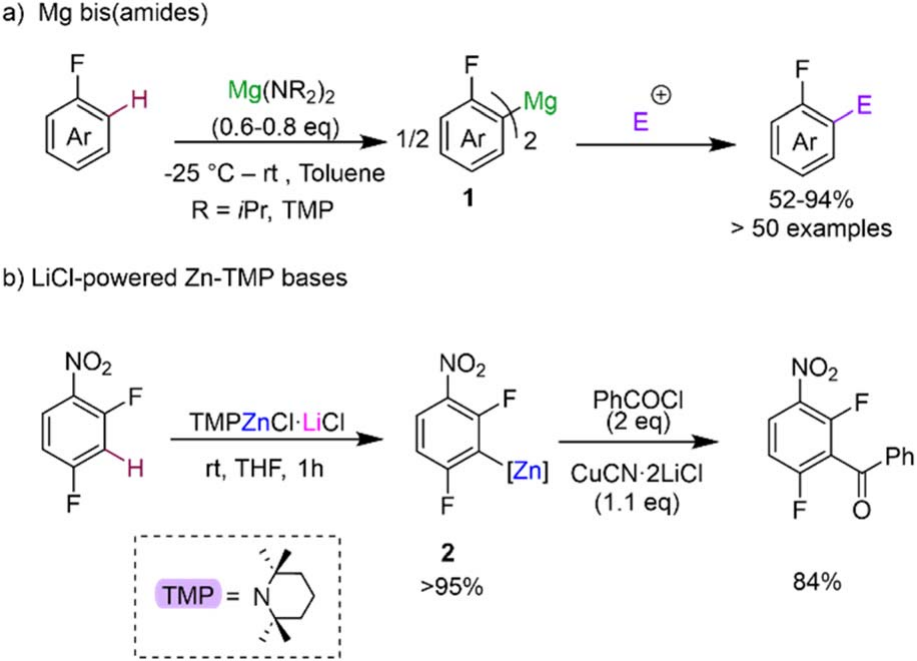
Magnesium and zinc bases for regioselective C–H metalation of fluoroarenes.

Deprotonative metalation constitutes one of the most powerful and widely used methods for the regioselective functionalisation of C–H bonds in fluoroarenes using main group metal reagents.^5^ However as mentioned above, one of the main pitfalls when using conventional Group 1 metal bases (such as alkyllithiums or Lochmann–Schlosser superbases) is the fragility of the metalated intermediates that can lead, in many cases, to decomposition and unwanted side reactions even when operating under cryogenic conditions.^5*b*^ To overcome this limitation, alternative metalating reagents have been developed using non-nucleophilic bases containing bulky basic amide groups in combination with less electropositive metals (*e.g.*, Mg, Zn, Al). Seminal advances in this aspect include Knochel's use of magnesium amides Mg(NR_2_)_2_ (NR_2_ = N*i*Pr_2_, TMP; TMP = 2,2,6,6-tetramethylpiperidide) for regioselective C–H magnesiation of a wide range of substituted fluoroarenes producing bis-aryl magnesium intermediates MgAr^F^_2_ (1) (Scheme 2a).^17^ This methodology boasts an impressive substrate scope (including perfluoroarenes and fluoropyridines) and tolerates sensitive functional groups such ester, amide, azide and oxazoline functionalities. Remarkably, most of these reactions can be carried out at room temperature in toluene solutions. While no trapping or characterisation of the metalated intermediates 1 has been reported, their electrophilic interception has been extensively studied, demonstrating the excellent versatility of this approach to furnish highly functionalised fluoroarenes. The same group has also developed highly chemoselective LiCl-powered Zn bases containing TMP groups, namely TMPZnCl·LiCl and TMP_2_Zn·2MgCl_2_·2LiCl.^12*a*,18^ These mild and efficient Zn bases can successfully promote direct Zn–H exchanges of fluoroarenes bearing sensitive functional groups such as nitro (as shown for 2,4-difluoronitrobenzene in Scheme 2b).^12*a*^ Zincated intermediates typified by 2 can participate directly in Negishi cross-couplings or undergo transmetalation with Cu salts to form the target aroylation products in high yields (Scheme 2b). Clososki has shown that both TMPMgCl·LiCl and TMP_2_Zn·2MgCl_2_·2LiCl can metalate a wide range of fluorinated nitriles, such as 3-fluorobenzonitrile, in THF solution under mild conditions (THF, room temperature, 0.5–1 h) avoiding competing addition reactions to these unsaturated substrates.^19^ Collectively these insightful synthetic studies showcase the vast potential of Mg and Zn bases as more selective alternatives to conventional organolithium or lithium amide methods for the metalation of fluoroarenes, generating more robust metalated intermediates *in situ* while operating at room temperature conditions. While from a mechanistic perspective the constitution of the active organometallic intermediates in these reactions remains uncertain, work assessing the constitution of Turbo Hauser bases (NR_2_)MgCl·LiCl and TMPZnBr·LiBr has revealed the involvement of kinetically activated lithium magnesiate and lithium zincate species respectively.^20^

**Fig. 1:**
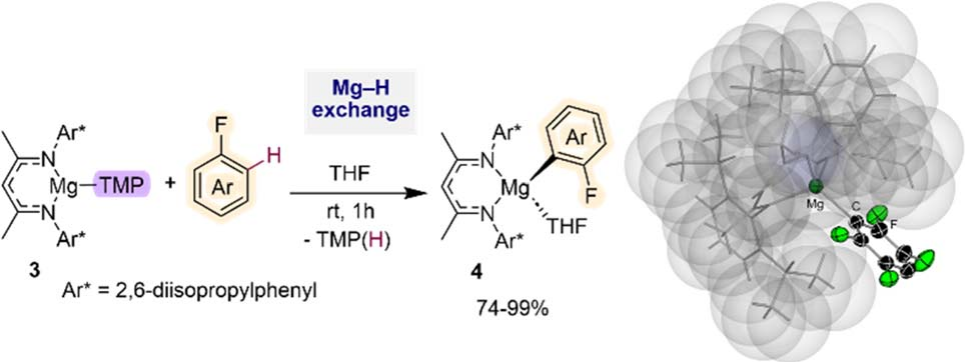
C–H magnesiation of fluoroarenes by [(^Dipp^Nacnac)Mg(TMP)] (3). Molecular structure of [(^Dipp^Nacnac)Mg(C_6_HF_4_)] using a space filling model for the β-diketiminate ligand.

Recently Hevia has employed specially designed β-diketiminate stabilized mononuclear Mg base [(^Dipp^Nacnac)Mg(TMP)] (3) (^Dipp^Nacnac = Ar*NC(Me)CHC(Me)NAr*; Ar* = 2,6-*i*Pr_2_–C_6_H_3_) for regioselective Mg–H exchange reactions of fluoroarenes (Fig. 1). Reactions with a range of substrates showed excellent yields for accessing the relevant metalated intermediates [(^Dipp^Nacnac)Mg(Ar^F^)] (4) (74–99% yield), subsequently used as precursors in Negishi cross-coupling reactions *via* transmetalation with ZnCl_2_.^21^ Arylmagnesiation products 4 could be isolated and fully characterised. Remarkably reactions could take place at room temperature, and intermediates 4 showed surprising thermal stability. Thus, even under forceful conditions (80 °C, 5 h), in the presence of benzyne trapping reagents, no evidence of decomposition was observed. This unexpected robustness of 4 has been rationalised in terms of the steric protection provided by the β-diketiminate ligand, providing steric shelter to the newly formed Mg–C bond (Fig. 1). Interestingly, replacing the TMP group by a more basic butyl group completely shuts down the metalation process, illustrating the kinetic attenuation of the metalating power of [(^Dipp^Nacnac)Mg(*n*Bu)(THF)]. In a related study, Hevia previously noted the enhanced kinetic basicity of the Mg amide 3 compared to the Mg-alkyl congener [(^Dipp^Nacnac)Mg(*n*Bu)(THF)] where the later forms simple coordination adducts with N-heterocyclic molecules, such as diazines, whereas the Mg amide 3 can regioselectively magnesiate these sensitive arenes.^21*b*^

**Fig. 2:**
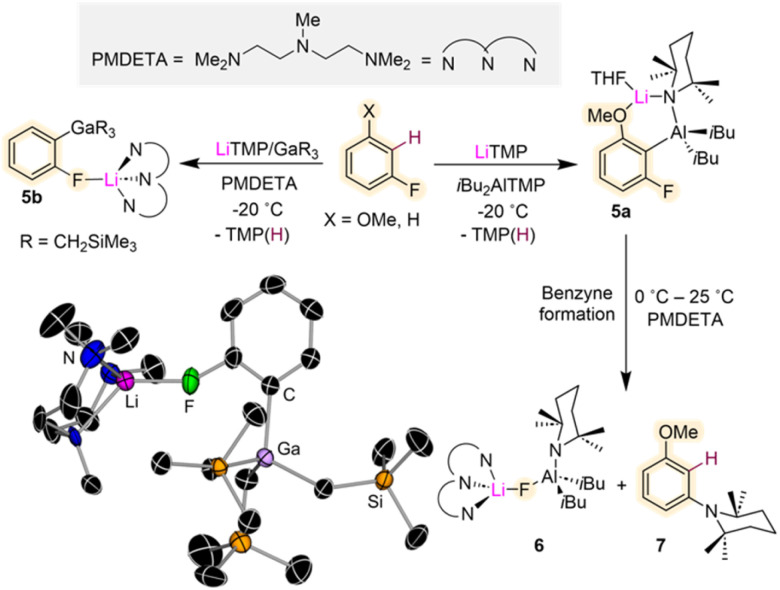
Contrasting Al *vs.* Ga Trans-Metal-Trapping (TMT) outcomes of reactions with fluoroarenes. Molecular structure of [2-Ga(CH_2_SiMe_3_)_3_-1-F-C_6_H_4_·Li(PMDETA)] (5b) resulting from the 1 : 1 reaction of fluorobenzene with LiTMP/Ga(CH_2_SiMe_3_)_3_/PMDETA in hexane.

While, as mentioned above, direct lithiation of fluoroarenes can be challenging Mulvey and Hevia have shown that using the utility amide LiTMP combined with an organometallic trap such as Al(TMP)*i*Bu_2_ or Ga(CH_2_SiMe_3_)_3_, can stabilise the relevant metalated intermediates and allow their structural characterisation (Fig. 2).^22^ This approach also known in the literature as Trans-Metal-Trapping (TMT) relies on the steric incompatibility between the metalating reagent (LiTMP) and the organometallic trap which precludes their co-complexation to form an ate complex. Instead, operating in tandem, these bimetallic mixtures exploit the strong basicity of the lithium amide with the stronger carbophilicity of the trapping agent (which traps and stabilizes the incipient anion generated *via* metalation) to enable functionalisation of aromatic substrates with high selectivity under mild reaction conditions.^23^ TMT studies using a LiTMP/Al(TMP)*i*Bu_2_ combination towards 3-fluoroanisole furnished alumination product [2-{(*i*Bu)_2_Al(μ-TMP)Li·THF}-3-fluoroanisyl] (5a) (Fig. 2). The bimetallic contacted ion pair structure of 5a was confirmed by X-ray crystallographic analysis. The scope of this reactivity could be extended to related fluoroarenes, although only moderate yields were obtained, and the relevant intermediates were found to decompose at room temperature. Studies investigating the possible decomposition products of these reactions revealed that at room temperature 5a eliminates LiAlF(TMP)*i*Bu_2_ (6) which can be isolated as a crystalline solid on adding the tridentate Lewis donor PMDETA (*N*,*N*,*N*′,*N*′′*N*′′-pentamethyldiethylenetriamine). NMR spectroscopic analysis of the reaction crude showed the concomitant formation of (3-methoxyphenyl)-2,2,6,6-tetramethylpiperidine (7) as a by-product resulting from the interception of the relevant benzyne species with amine TMP(H) (present in solution from the initial lithiation reaction)^22^ (Fig. 2). Contrastingly when the same approach is employed using Ga(CH_2_SiMe_3_)_3_ as a metal trap, more robust intermediates can be obtained as shown in Fig. 2 for gallation of fluorobenzene which furnishes the gallate [2-Ga(CH_2_SiMe_3_)_3_-1-F-C_6_H_4_·Li(PMDETA)] (5b). While the metalation reaction needs to be performed at −78 °C, 5b is stable in solution at room temperature and it can engage in further functionalisation reactions as for example Pd-catalysed aroylation reactions. The greater stability of 5b*vs.*5a is attributed to the more fluorophilic character of Al *vs.* Ga.

**Scheme 3:**
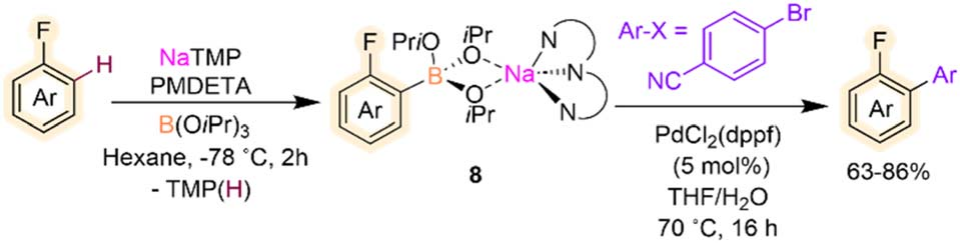
Sodium-mediated deprotonative C–H borylation of fluoroarenes using NaTMP/PMDETA/B(O*i*Pr)_3_ with subsequent Suzuki–Miyaura cross-coupling.

Related metalation strategies have been developed using NaTMP/PMDETA/B(O*i*Pr)_3_ combinations, which tolerate fluoroarenes including fluorobenzene, 4-fluoroanisole and 4-fluorotoluene.^24^ Reactions need to be carried out at −78 °C, and the presence of PMDETA is crucial in order to de-aggregate the sodium amide and enhance its solubility and kinetic reactivity. For these substrates, regioselective *ortho*-sodiation to the fluorine substituent occurs followed by fast trapping with B(O*i*Pr)_3_ furnishing sodium borates [(PMDETA)NaB(Ar^F^)(O*i*Pr)_3_] (8). These intermediates are stable at room temperature and can be directly used in Pd-catalysed Suzuki–Miyaura cross-couplings to give desired bis(aryl) products in good yields (Scheme 3). This example draws attention to the initial sodiation and subsequent trapping of the fluoroarene however the borylation and silylation of fluoroarenes *via* C–F functionalisation using main group metal strategies has been reviewed previously and lies outside the scope of this Perspective.^25^

**Fig. 3:**
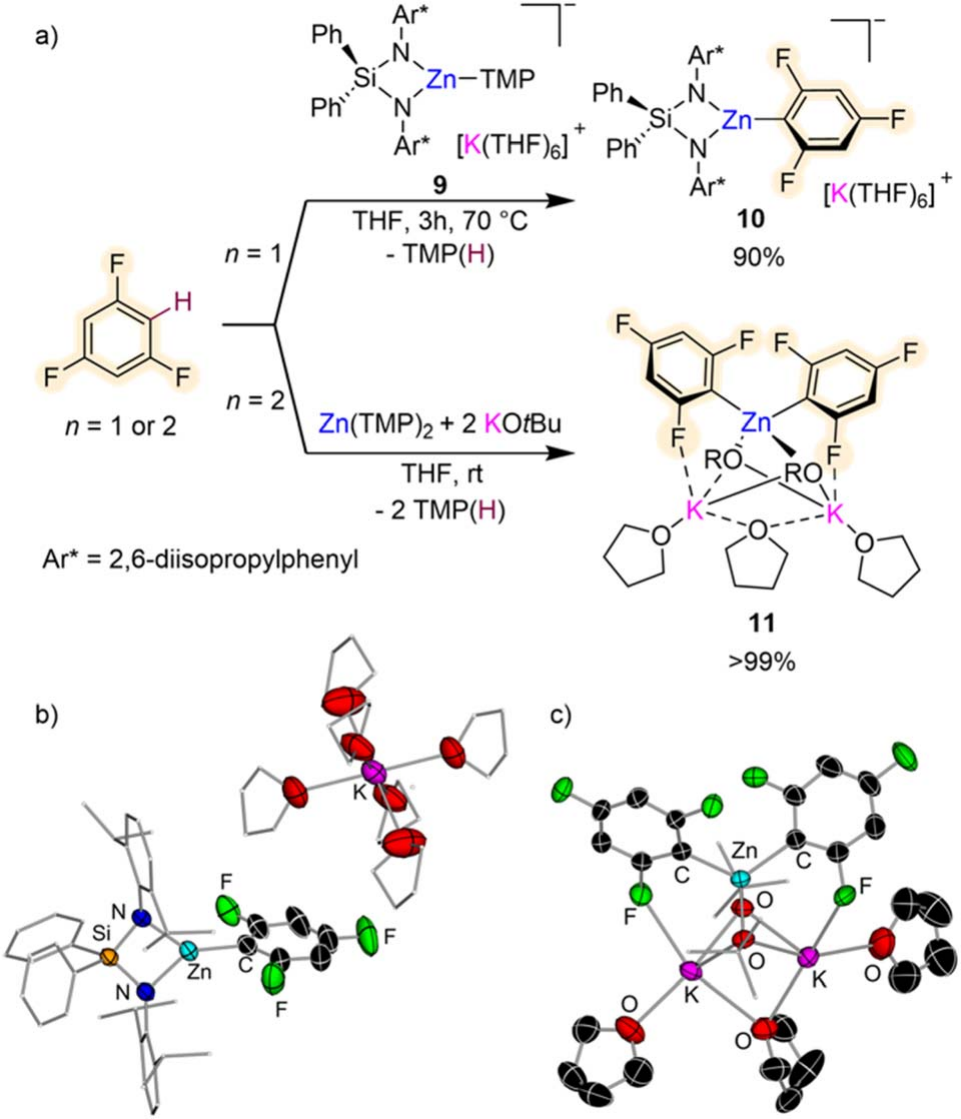
(a) Comparative reactivity of potassium zincates towards the C–H zincation of 1,3,5-trifluorobenzene. (b) Crystal structures of [{Ph_2_Si(NAr*)_2_Zn(C_6_H_2_F_3_)}^–^{K(THF)_6_}^+^] (10) and (c) [(THF)_3_K_2_Zn(C_6_H_2_F_3_)_2_(O*t*Bu)_2_] 11.

Other bimetallic systems which have shown promise for fluoroarene metalation are potassium zincates. Combining a bulky bis(amide) {Ph_2_Si(NAr*)_2_}^2−^ (Ar* = 2,6-diisopropylphenyl) and a reactive one-coordinate TMP ligand, Hevia has developed the K/Zn heteroleptic base [{Ph_2_Si(NAr*)_2_Zn(TMP)}^–^{K(THF)_6_}^+^] (9) (Fig. 3a) for regioselective zincation of fluoroarenes.^26^ This special ligand set allows for trapping and characterisation of the organometallic intermediate from these Zn–H exchange processes as shown in Fig. 3b for formation of [{Ph_2_Si(NAr*)_2_Zn(C_6_H_2_F_3_)}^–^{K(THF)_6_}^+^] (10) which can be isolated in 90% yield by reacting equimolar amounts of 9 and 1,3,5-trifluorobenzene for 3 h at 70 °C in THF. Both compounds 9 and 10 exhibit solvent-separated ion-pair structures, with potassium hexa-coordinated by THF whereas Zn displays a trigonal planar geometry, bound to the bidentate silylbis(amido) ligand and a terminal amide (or aryl) group (Fig. 3b). This approach can be extended to a wide range of fluoroarenes including those containing highly sensitive NO_2_ groups with reaction conditions varying from −40 °C to 70 °C, depending on the degree of activation of the substrate (in terms of p*K*_a_ values). Fluorobenzene only reacted sluggishly (17% after 24 h at 70 °C). Preliminary reactivity studies have also shown that these intermediates can undergo Pd-catalysed C–C bond forming processes with bromoarenes. Remarkably dizincation of 1,2,4,5-tetrafluorobenzene can be achieved by using 2 equivalents of 9. It should be noted that Zn(TMP)_2_ on its own fails to metalate these substrates and also that when ultrasensitive nitrofluoroarenes were reacted with Mulvey's potassium zincate [(PMDETA)KZn(TMP)Et_2_],^27^ a complex mixture of decomposition products was observed. These findings suggest that key for the success of this approach is the combination of a sterically demanding bis(amide) supporting ligand, which provides stability to the fragile fluoroaryl fragments in the metalated intermediates; and a terminal, one-coordinate, kinetically ate-activated TMP basic site on 9, which enables direct zincation of the substrates.

Enhancement of the metalating power of Zn(TMP)_2_ towards fluoroarenes has recently being accomplished by addition of two equivalents of KO*t*Bu (Fig. 3a). This approach extends the concept of using alkali-metal alkoxides as additives for the development of more powerful superbasic reagents, pioneered independently by Lochmann and Schlosser,^28^ to zinc amide chemistry. Remarkably direct zincation of 1,3,5-trifluorobenzene can be achieved in just 10 minutes at room temperature furnishing higher order potassium zincate [(THF)_3_K_2_Zn(C_6_H_2_F_3_)_2_(O*t*Bu)_2_] (11) (Fig. 3a).^29^

Interestingly, both TMP groups are active towards the Zn–H exchange and the two equivalents of KO*t*Bu are integrated within the constitution of the metalation product. While Zn coordinates to the C atom of the substrate that has experienced the metalation, K prefers to bind to one of the *ortho*-F atoms (Fig. 3c). These distinct coordination modes are thought to contribute to the overall stability of 11 which does not undergo decomposition even under refluxing conditions (70 °C). Mechanistic investigation on how this Zn(TMP)_2_/2KO*t*Bu bimetallic mixture operates revealed that the first equivalent of potassium alkoxide undergoes co-complexation with the Zn amide to form potassium zincate [(THF)_*x*_KZn(TMP)_2_(O*t*Bu)] enabling kinetic activation of both TMP groups which can now react with two equivalents of substrate; whereas the second alkoxide equivalent stabilizes metalated intermediate 11 preventing ligand redistribution. This approach was found to be versatile in terms of substrate scope allowing also efficient zincation of non-activated flouroarenes (such as fluorobenzene or 1-fluoronaphthalene) while displaying excellent chemo- and regioselectivities. However, when nitrofluoroarenes were tested intractable decomposition products were obtained. Higher order potassium zincate intermediates such as 11 are stable and can be isolated as solids as well as prepared *in situ* for electrophilic interception reactions such as Pd-catalysed Negishi cross-couplings or Cu catalysed allylations/aroylations.

**Scheme 4:**
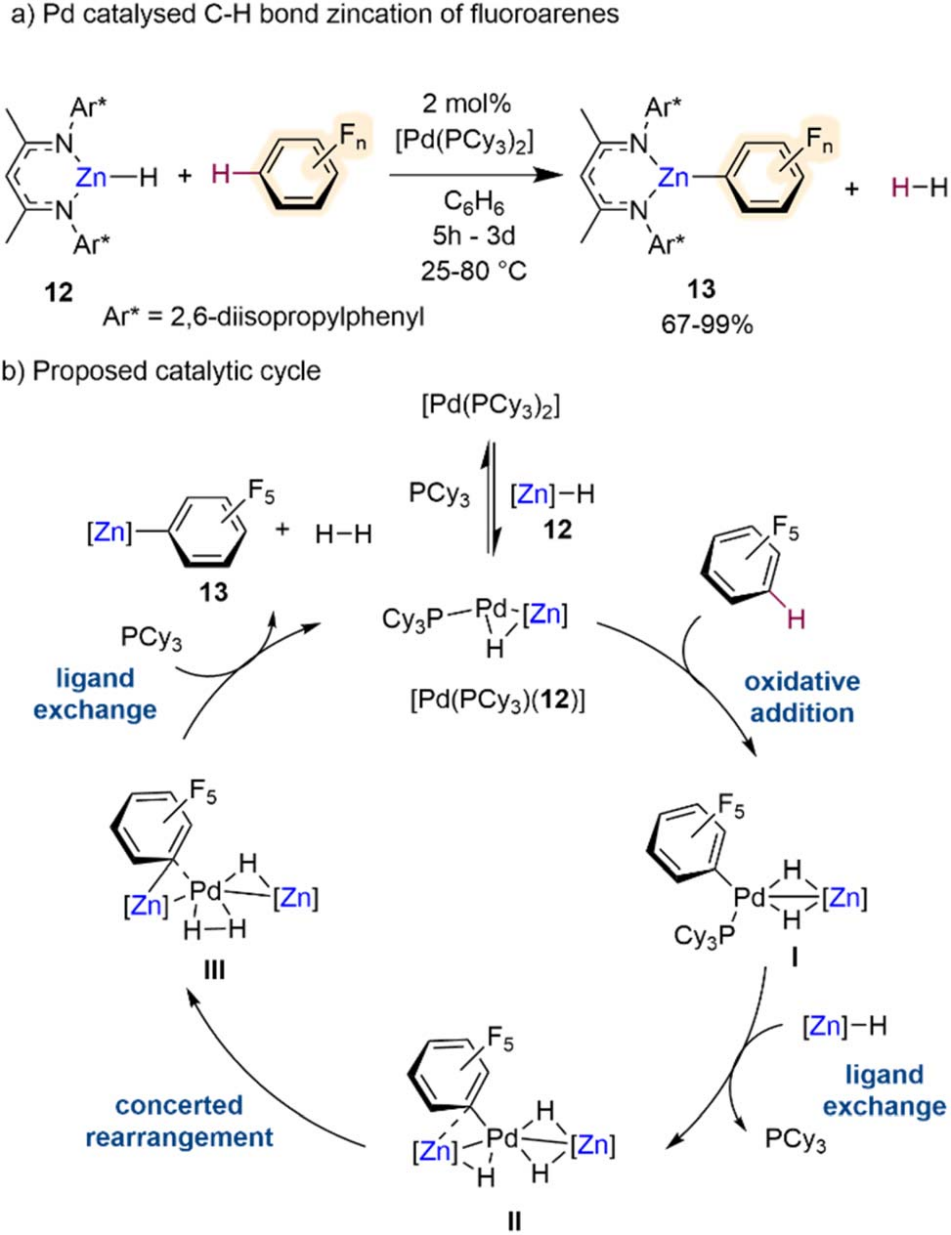
(a) C–H bond zincation of fluoroarenes *via* β-diketiminate supported Zn-hydride catalysed by [Pd(PCy_3_)_2_]. (b) Proposed catalytic cycle *via* heterobimetallic Zn–Pd intermediates.

Outwith these potassium-mediated-zincation strategies, Crimmin has recently developed an elegant method for C–H bond zincation of fluoroarenes using Pd catalysis.^30^ Combining monomeric zinc hydride [(^Dipp^Nacnac)ZnH] (12)^31^ with catalytic amounts of [Pd(PCy_3_)_2_] the chemo- and regioselective transformation of C–H bonds to C–Zn bonds in a wide range of fluoroarenes has been realised furnishing Zn aryl intermediates [(^Dipp^Nacnac)ZnAr^F^] (13) (Scheme 4a) with concomitant elimination of dihydrogen by-product. The chemoselectivity of this process is noteworthy considering that the Pd catalyst on its own, in the absence of 12, promotes preferentially the competing C–F bond activation of some of the substrates investigated. Interestingly, this Zn/Pd system offers complementary chemoselectivities to those reported by the same group using aluminium(III) dihydride [(^Mes^Nacnac)AlH_2_] (Mes = 2,4,6-trimethylphenyl) in combination with catalytic amounts of [Pd(PCy_3_)_2_], which allows for C–F bond alumination of fluoroarenes including fluorobenzene.^32^ Regarding the substrate scope of this Zn/Pd approach, activated substrates with high fluorine content react under milder conditions and can tolerate halogen, trifluoromethyl and methoxy functional groups. However, lowering the fluorine content leads to sluggish reactivity, for example the C–H zincation of fluorobenzene afforded less than 30% product after 3 days at 80 °C. This limitation can be overcome by using excess substrate (10 equivalents). Insightful mechanistic studies combining kinetic investigations with DFT calculations and the trapping of key reaction intermediates, revealed that these transformations involved formation of heterobimetallic Pd/Zn species, and it is underpinned by special cooperation between the two distinct metal partners (Scheme 4b). Complex [Pd(PCy_3_)(12)] was identified as a key catalytic intermediate. Oxidative addition of the fluoroarene, in this case C_6_F_5_H, across the 14-electron Pd^0^ forms complex I with a modest calculated stabilisation of the product. A ligand exchange reaction between complex I and a further equivalent of Zn hydride 12 gives complex II with liberation of a phosphine ligand. A concerted rearrangement of complex II to complex III occurs *via* a simultaneous migration of the perfluorinated σ-aryl ligand from Pd to Zn and a reductive coupling of the hydride ligands. This rearrangement requires a reduction in the formal oxidation state in Pd from Pd^II^ to Pd^0^. The catalytic cycle is then closed *via* a further ligand exchange process of complex III by coordination of PCy_3_ with concomitant liberation of H_2_ and the desired product of C–H bond zincation 13 to reform [Pd(PCy_3_)(12)]. Whilst not discussed thoroughly in this catalytic cycle, the authors attribute the primary role of the β-diketiminate ligand to provide kinetic stability to the reactive intermediates in the catalysis and the Zn hydride complex. Hence, the ligand–metal cooperativity is of paramount importance to the C–H zincation of the fluoroarenes along with the bimetallic cooperativity between Zn and Pd.

## Main group metal-mediated C–F bond activations of fluoroarenes

As previously mentioned, the large electronegativity difference between the carbon atom and the small but very electronegative fluorine (*r*_W_ = 1.47 Å and *χ* = 4) make C–F bonds polar and exceptionally strong.^1*c*,6,7^ Moreover, addition of a F atom to an aromatic molecule has shown to drastically change the properties of the whole molecule, resulting in an increase strength of the adjacent C–H or C–M bond as well decreasing the strength of other C–F bonds present.^1*c*,6,33^ This results in a more challenging C–F activation of partially fluorinated arenes compared to perfluorinated substrates, that in some cases can be accompanied by the kinetically more favourable C–H activation. Although C–F activation has predominantly been investigated using precious transition metal complexes,^10^ several developments over the past decade have demonstrated the large potential of main-group metal complexes to split these strong σ-bonds.^34^ The vast majority of these examples rely on the use of low valent metal complexes in which ligand/metal or bimetallic cooperativity play a major role. A distinct feature of the systems employed to perform these selected C–F bond activations is the use of highly sterically demanding ligands, carefully designed to provide sufficient stability to the low valent metal centre without compromising their reactivity.^16*a*^

Monovalent Al monomer [(^Dipp^Nacnac)Al] (14) (^Dipp^Nacnac = Ar*NC(Me)CHC(Me)NAr*; Ar* = 2,6-*i*Pr_2_–C_6_H_3_), first synthesized by Roesky and described as a stable Al analogue of a N-heterocyclic carbene,^35^ illustrates nicely how sustainable low valent main group metal complexes can mimic the reactivity of precious transition metal systems. Containing lone pairs of electrons on the nitrogen atoms of the ligand that poorly overlap with the Al orbitals, combined with the low electronegativity of Al, 14 can act both as a good nucleophile and a good Lewis acid. Being stabilised by a bulky β-diketiminate ligand, 14 has shown an impressive potential to activate a variety of E–H bonds (either hydridic or protic; E = B, C, Si, N, P, O) as well as H_2_ or Sb–Sb bonds *via* a formal oxidative addition step.^36^

**Scheme 5:**
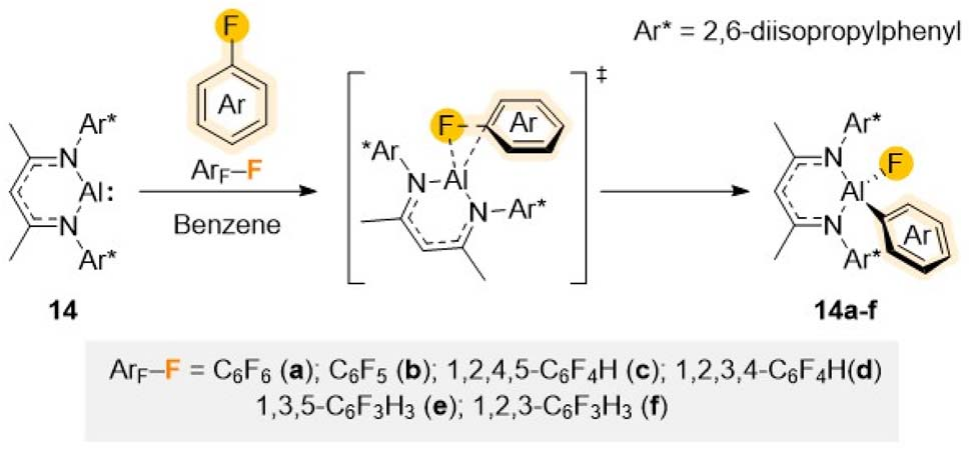
Structure of Al(i) reagent 14 and proposed mechanism for its reaction with fluoroarenes Ar–F (a–f) *via* a concerted S_N_Ar mechanism.

The ability of 14 to promote C–F bond activation of a wide range of perfluorinated substrates has been demonstrated separately by the groups of Crimmin and Nikonov.^37^ Reaction conditions (time and temperature) vary depending on the degree of activation of the substrates, increasing as the number of F atoms decreases in the substrate. Mechanistic investigations, combining DFT calculations with kinetic studies and trapping of key reaction intermediates, support a transition metal–like concerted oxidative addition of the fluoroaromatic substrate to the Al centre as shown in Scheme 5.^37*b*,38^

Recent work by Kretschmer has also revealed that (Cp*Al)_4_ (15), first synthesised by Schnöckel over twenty years ago,^39^ can also activate C–F bonds of perfluorotoluene, pentafluoropyridine as well as 1,2,3,4-tetrafluoro-, pentafluoro- and hexafluorobenzene.^40^ Reactions occur with excellent regioselectivities although relative forcing conditions (90 °C, with reaction times spanning 15 minutes to 5 days).

**Scheme 6:**
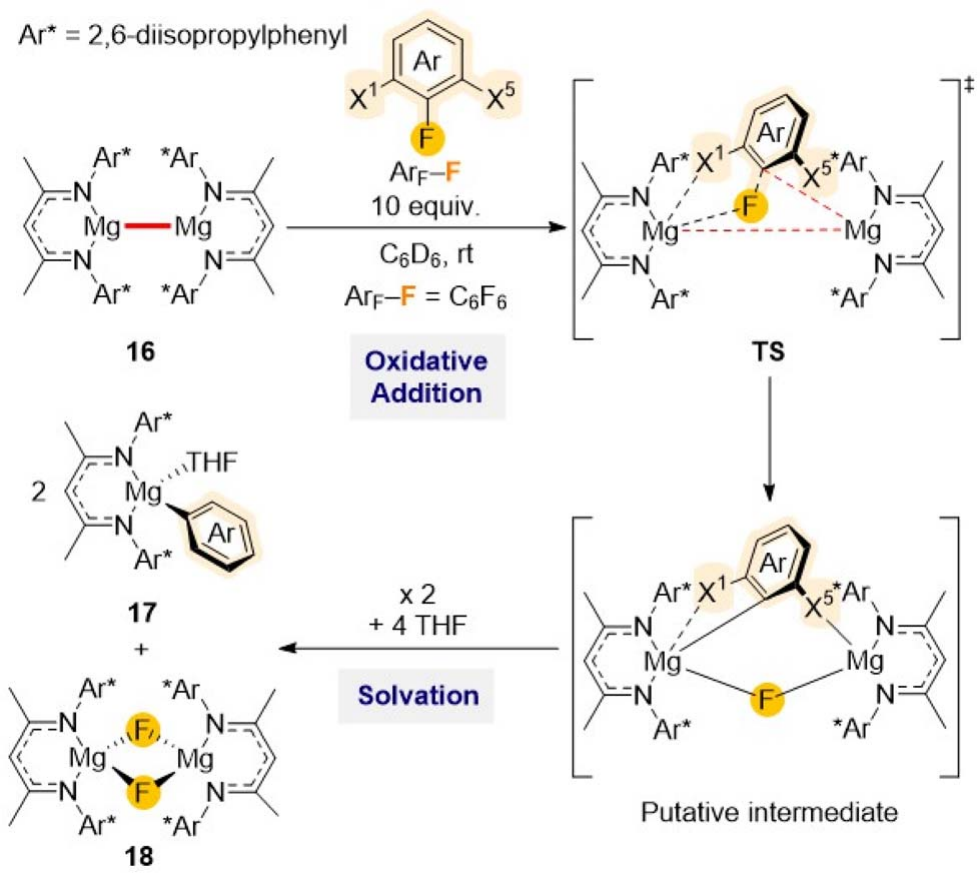
Reaction of di-nuclear Mg(i) complex 16 with C_6_F_6_ and proposed mechanism for formation of Mg(ii) products 17 and 18.

Low oxidation state Mg(i) homobimetallic complex [(^Dipp^Nacnac)Mg–Mg(^Dipp^Nacnac)] (16)^15*a*^ has also shown potential for C–F bond activation reactions as demonstrated by Crimmin when investigating reactivity of 16 towards a series of per- and partially-fluorinated arenes. Reactions occur with formation of new Mg–C and Mg–F bonds as illustrated in Scheme 6 for C_6_F_6_, furnishing the Mg(ii) aryl [(^Dipp^Nacnac)Mg(THF)(C_6_F_5_)] (17) and Mg(ii) fluoride [(^Dipp^Nacnac)Mg(μ-F)(THF)_2_] (18).^41^ This approach was probed with a wide range of substrates finding in most cases good control of the regioselectivity, with the activation always occurring on the C–F bond with at least one *ortho*-fluorine substituent and leaving the C–H bonds untouched.

Further mechanistic investigations using DFT calculations revealed that this C–F bond activation process is unlikely to proceed through radical intermediates.^42^ Alternatively a concerted pathway seems more feasible, involving the initial weak coordination of the substrate to the dinuclear Mg(i) complex *via* multiple non covalent interactions, followed by rotation around the Mg–Mg bond to access the more reactive conformer with a sterically accessible Mg–Mg site for addition of the C–F bond. This is followed by the formation of a six-membered intermediate which ultimately form the C–F activation products 17 and 18 (Scheme 6). Moreover, steric effects were assessed by comparing β-diketiminate Mg(i) complexes containing different substituents at the N atom. This study showed that an increase in steric hindrance around the Mg–Mg bond contributes significantly to decreased reaction rates. DFT studies also highlighted the crucial role of the Mg⋯F_*ortho*_ interactions in influencing Gibbs activation energies for the transition state (TS). These studies reveal that C–F bonds adjacent to one or multiple fluorine substituents are both thermodynamically and kinetically activated and that the substitution of a fluorine with a hydrogen at the X_1_ position (Scheme 6) precludes the substrate from interacting with the Mg centre, increasing the energy barrier for the TS. More recently this reactivity has been extended to room temperature defluorination of polyfluoroalkyls such as poly(tetrafluoroethylene) (PTFE) using [(^Mes^Nacnac)Mg–Mg(^Mes^Nacnac)] (19) (Mes = 2,4,6-trimethylphenyl) in combination with 4-(dimethylamino)pyridine (DMAP) generating molecular magnesium fluoride which can be used as a viable fluorinating agent.^43^

An interesting example of Mg-mediated C–F bond activation of the most challenging substrate, fluorobenzene, has been reported by Harder employing [(^Dipep^Nacnac)_2_Mg_2_(C_6_H_6_)] (20) (^Dipep^Nacnac = Ar′′NC(Me)CHC(Me)NAr′′; Ar′′ = 2,6-diisopentyl–C_6_H_3_) which contains a formally reduced benzene {C_6_H_6_}^2−^ dianion and a particularly sterically encumbered β-diketiminate support. While the reaction is slow and harsh reaction conditions are employed (5 days, 100 °C), formation of the expected oxidative addition products [(^Dipep^Nacnac)Mg(C_6_H_5_)] (21) and [(^Dipep^Nacnac)MgF] along with benzene is observed.^44^ While 20 acts formally as a 2-electron donor, the authors cautiously state that Brønsted base reactivity or complex decomposition prior to reaction cannot be ruled out.

**Scheme 7:**
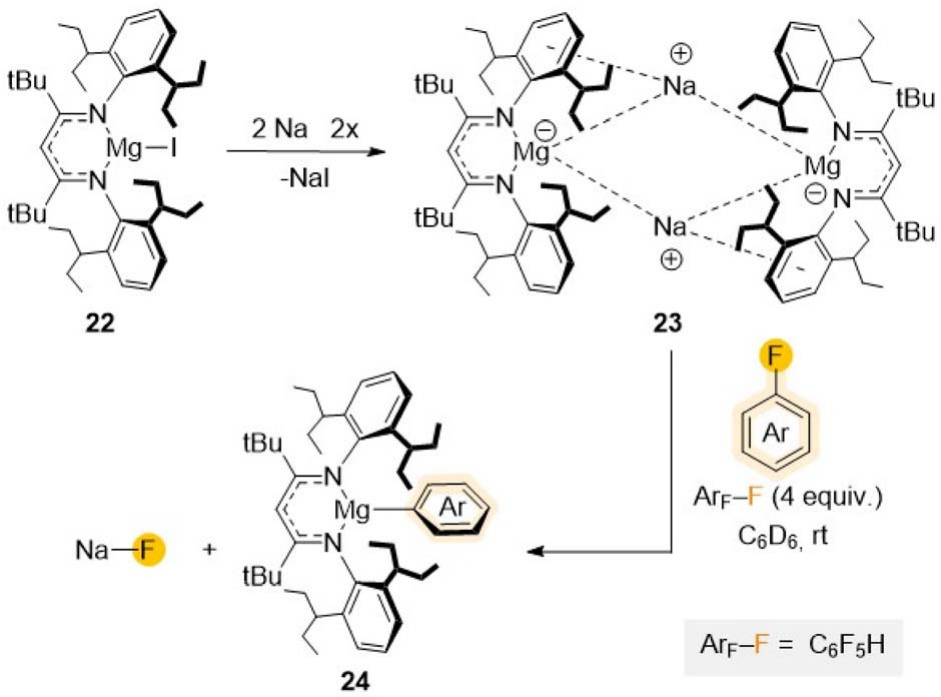
Synthesis of Mg(0) complex 23 and reactivity towards C_6_F_5_H to give C–F activation product 24.

Increasing even further the steric hindrance around the Mg centre, a new Mg(i) complex [(^Dipep^L)MgI] (22) (^Dipep^L = Ar′′NC(*t*Bu)CHC(*t*Bu)NAr′′; Ar′′ = 2,6-diisopentyl–C_6_H_3_) presenting two additional *t*Bu groups in the ligand backbone was reported in 2020 with the intention to stabilize potential Mg(i) radical species (Scheme 7).^45^ Reduction of 22 with Na/NaCl led to the formation of a unique magnesyl sodium complex {[(^Dipep^LMg^−^)][Na^+^]}_2_ (23) with two anionic {(^Dipep^L)Mg}^−^ fragments bridged by two Na^+^ cations.^46^ Remarkably, this formally Mg(0) species (23) displays exceptional redox reactivity towards fluorobenzene furnishing Mg(ii) aryl [(^Dipep^L)Mg(C_6_H_5_)] (24) with concomitant elimination of NaF. Contrasting with previous studies using Mg(ii) complex 20, containing a strongly reducing {C_6_H_6_}^2−^ anion, which required the use of forcing reaction conditions (5 days, 100 °C),^44^ the C–F bond activation of fluorobenzene using 23 occurs rapidly at room temperature (10 min, 20 °C), highlighting the enormous difference in reactivity on these two related Mg complexes where Mg displays different oxidation states.^46^

**Scheme 8:**
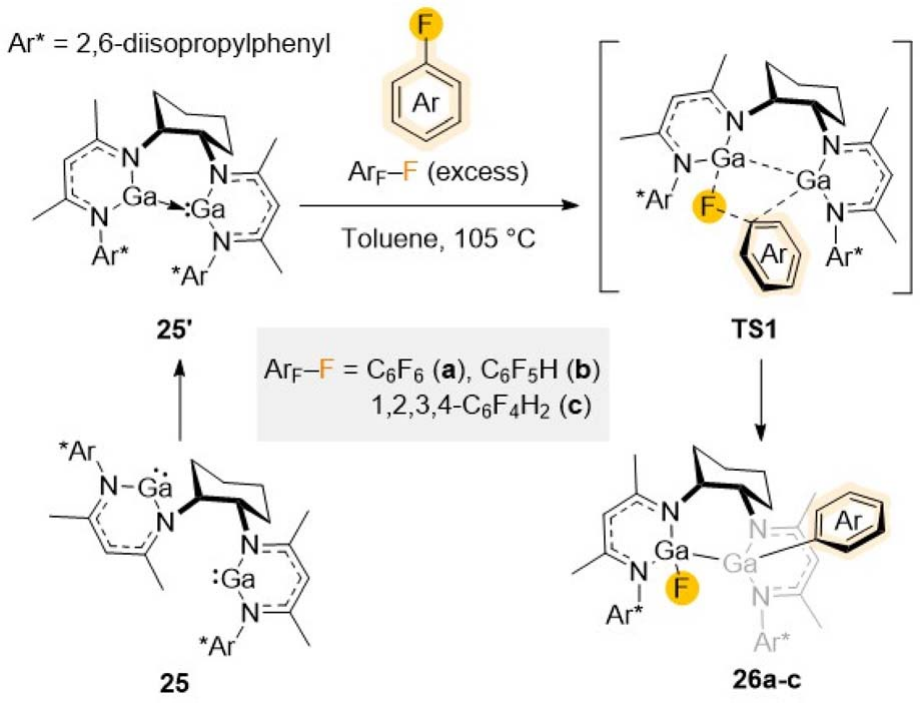
Reaction of 25 with fluoroarenes (Ar_F_–F a–c) to give C–F activation products 26a–c.

Exploiting bimetallic cooperativity, Kretschmer has developed dinuclear gallanediyl complex 25 featuring two separate dicoordinate Ga(i) centers used to activate C–F bonds (Scheme 8).^47^ Containing a specially designed bis-β-diketimine ligand, each Ga in 25 possesses a lone pair of electrons and a vacant orbital being reminiscent of an N-heterocyclic carbene. Reactivity studies with C_6_F_6_ (a), C_6_F_5_H (b), and 1,2,3,4-tetrafluorobenzene (c) illustrate the ability of 25 to promote regioselective C–F bond activation of these substrates in quantitative yields *via* a cooperative bimetallic mechanism (Scheme 8).

Following the same trend already discussed for other examples above, refluxing conditions (toluene, 105 °C) are required and reaction times (ranging from 1 to 48 h) depend on the degree of fluorination of the substrate furnishing 26a–c. Displaying a bimetallic Ga(ii) motif, 26a–c contain a Ga–Ga bond, with one Ga coordinated to a F atom and the remaining Ga bonded to the aryl group (Scheme 8). Demonstrating the importance of Ga/Ga cooperation in 25, greater performances and milder reaction conditions were observed than when using its mononuclear Ga(i) analogue [(^Dipp^Nacnac)Ga]. DFT calculations showed that while insertion of the fluoroarene's C–F bond into the two individual Ga centres in 25 seems to be kinetically unfavoured, the reaction of the Ar^F^–F with its isomeric form 25′, where both metals are in close proximity to each other, forming a direct Ga–Ga interaction, can proceed effectively. The reaction proceeds *via*TS-1 (Scheme 8), followed by concerted cleavage of the C–F bond cleavage and Ga–C, Ga–F and Ga–Ga bond formation.

**Scheme 9:**
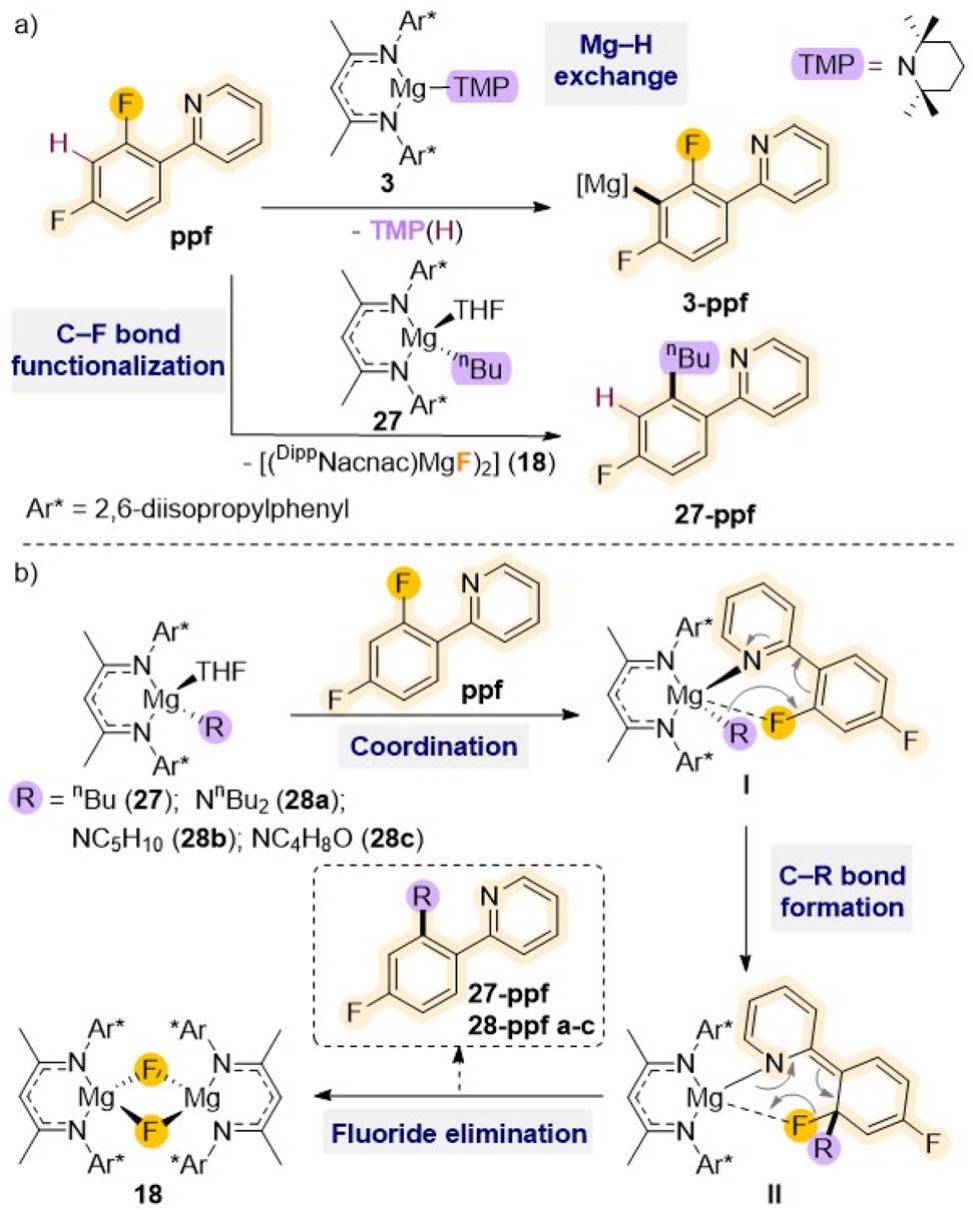
(a) Reactivity of β-diketiminate stabilised Mg(ii) complexes 3 and 27 with ppf: deprotonative metalation *vs.* C–F bond activation; (b) general proposed reaction mechanism for Mg-mediated C–F bond alkylation/amination of ppf.

Other studies employing Mg(ii) complexes supported by a similar sterically demanding β-diketiminate ligand have shown their ability to regioselectively functionalised fluoroarenes such as 2-(2,4-difluorophenyl)pyridine (ppf) *via* Mg–H exchange (*vide supra*) or C–F bond alkylation (Scheme 9a).^21*a*^ Thus, while using amide complex [(^Dipp^Nacnac)Mg(TMP)] (3) allows for magnesiation at the C3 atom of the fluoroaryl ring, the reaction with the *n*-butyl derivative [(^Dipp^Nacnac)Mg(*n*Bu)THF] (27) results in almost quantitative formation of the fluoride complex [{(^Dipp^Nacnac)MgF(THF)}_2_] (18) together with the alkylation product 27–ppf (Scheme 9a).

Mechanistic investigations suggest that initial precoordination of the pyridine nitrogen atom to the Mg centre (I in Scheme 9b) seems to be the key to initiate the reaction, as other activated perfluoroarenes where this type of pre-coordination is not possible such as C_6_F_6_ or C_6_F_5_H fail to react with 27. This initial step brings the *ortho* C–F bond in close proximity to the Mg centre activating the molecule for subsequent addition of the *n*Bu group to the benzene ring. Ultimately, this allows formation of a new C–C bond (II in Scheme 9b), concomitant to the cleavage of the C–F bond and elimination of the fluoride complex 18. This approach has been extended to related β-diketiminate Mg(ii) complexes containing nucleophilic amide groups such as dibutylamide, piperidide or morphilide, which allows for efficient C–F to C–N bond transformation in ppf and 2-fluoropyridine under mild reaction conditions.^48^ These reactions are also favoured by the precipitation of fluoride complex 18. Interestingly these C–F bond cleavage/C–N bond formations occur regioselectively leaving adjacent C–H bonds intact.

Heterobimetallic complexes have also shown excellent capabilities for activation of C–F bonds enabled by bimetallic cooperativity. Crimmin has also made key advances in this field by utilizing a series of new heterobimetallic complexes containing polar M–M′ bonds [(^Dipp^Nacnac)M–M′(^Dipp^Nacnac)] (M = Mg, M′ = Zn (29), Al (30); M = Zn, M′ = Al (31)). The reactivity of these compounds were assessed and compared with those of homobimetallic Mg(i) complex 16 which allowed for an evaluation on how changes in polarization of the M–M′ bond can affect their reactivity towards fluoroarenes.^41^ Experimentally, contrasting with 16, it was found that while Mg–Al (30) and Zn–Al (31) complexes are inert towards perfluorinated substrates, bimetallic Mg–Zn (29) can activate 2-Py-C_6_F_5_ to generate [(^Dipp^Nacnac)Zn(2-Py-C_6_F_4_)] (32) and magnesium fluoride 18. DFT studies showed that while introducing polarity in the M–M′ bond can influence the selectivity of addition, it does not lead to faster reaction rates. Evidencing the importance of steric congestion, the more polar nature of the M–M′ bonds in these complexes leads to a shorter M–M′ distance (compared to that in 16), which overall hinders the approach of the fluoroarene to the metal centres to allow for the C–F bond cleavage.^42^

**Scheme 10:**
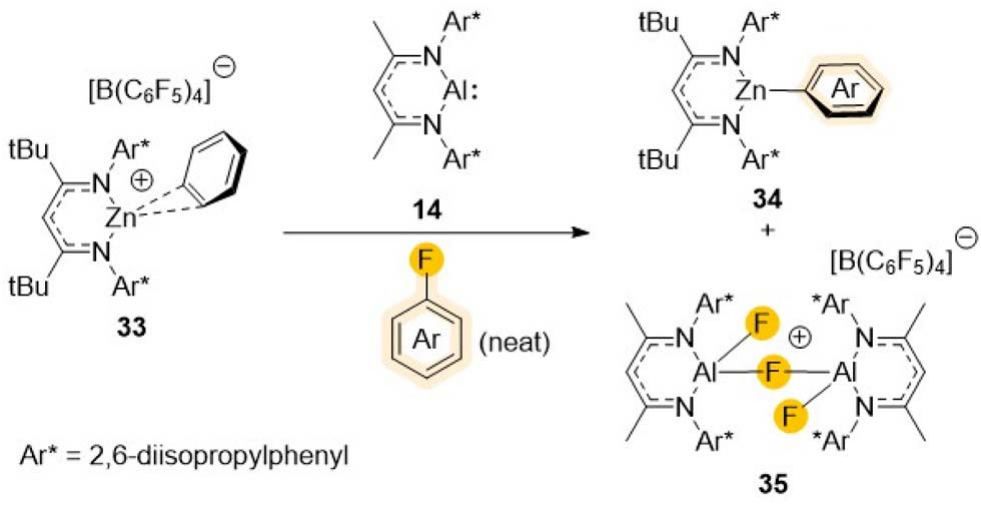
Cationic heterobimetallic complex 33 and an example of its reactivity towards fluorobenzene.

Looking into alternative ways to boost the reactivity, Harder has elegantly demonstrated that, contrasting with the inertness of [(^Dipp^Nacnac)Al] (14) towards fluorobenzene,^36^ the addition of [{(^Dipp^L)Zn·C_6_H_6_}^+^{B(C_6_F_5_)_4_}^–^] (33) (^Dipp^L = Ar*NC(*t*Bu)CHC(*t*Bu)NAr*; Ar* = 2,6-*i*Pr_2_–C_6_H_3_) to a solution of 14 in fluorobenzene allows for facile cleavage of its C–F bond furnishing [(^Dipp^L)ZnAr] (34) (Ar = Ph), and [{(^Dipp^Nacnac)Al(F)(μ-F)(F)Al(^Dipp^Nacnac)}^+^{B(C_6_F_5_)_4_}^–^] (35) (Scheme 10).^49^

Mechanistic investigations that also included DFT calculations revealed that the reactivity of this bimetallic system is synergic in origin, since none of the single components of this bimetallic combination can react with fluorobenzene on their own. Interestingly despite the facile C–F bond cleavage of the solvent, the C–F bonds of the B(C_6_F_5_)_4_^–^ anion are left intact. This has been attributed to the steric bulk of the latter which prevents formation of a (^Dipp^L)Zn^+^⋯B(C_6_F_5_)_4_^–^ contact ion-pair, whereas fluorobenzene can form the π-complex (^Dipp^L)Zn^+^·(π-C_6_H_5_F).^50^ The formation of this complex appears to be integral for the observed reactivity, allowing for the fluorobenzene activation towards 1,2 nucleophilic addition of electron rich [(^Dipp^Nacnac)Al] (14) to one of its C=C bonds to form an unusual intermediate where the Ph ring is dearomatized and can subsequently rapidly undergo rearomatisation providing the energy needed for the C–F bond cleavage.

**Scheme 11:**
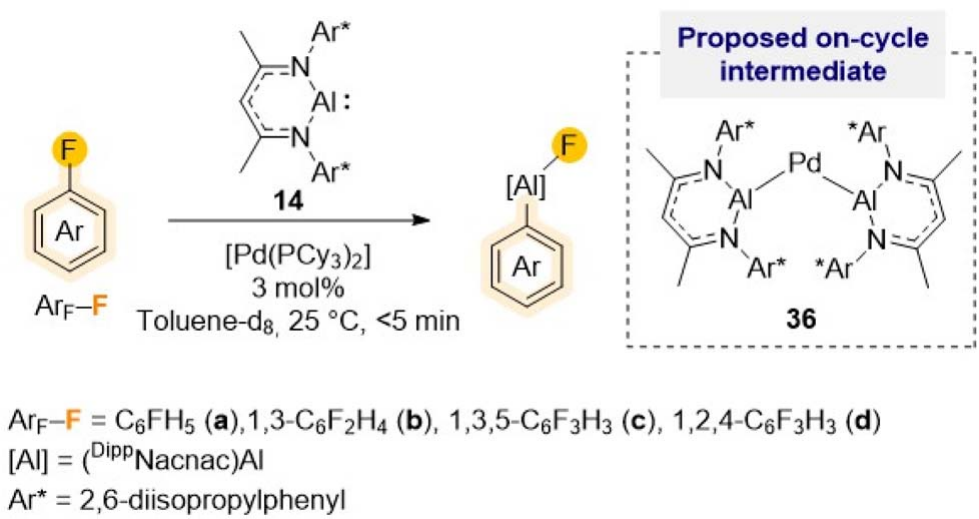
Palladium-catalysed C–F alumination of fluoroarenes using Roesky's Al(i) (14) and [Pd(PCy_3_)_2_] as pre-catalyst.

As alluded to above, fluorobenzene is a particularly challenging substrate for C–F bond activation reactions. Many other main-group metal mediated protocols, with the exception of the high reactivity shown by Mg(0) complex 23 (Scheme 7),^44^ require the use of harsh reaction conditions, irradiation or transition-metal catalysis.^43,51^ For example, using [Pd(PCy_3_)_2_] as pre-catalyst, efficient and selective C–F alumination of fluorobenzene can be achieved using Roesky's Al(i) complex 14 in just 5 min while operating at ambient temperature (Scheme 11).^51*c*^ Combining experimental studies with DFT calculations, mechanistic insights have been gained implying the involvement of heterobimetallic species such as {Pd[(^Dipp^Nacnac)Al]_2_} (36) as on-cycle intermediates during the catalysis. Interestingly, Crimmin has also shown that C–F bonds can be transformed into C–Al bonds using four coordinate diketaminate Al(iii) dihydrides as efficient terminal reductants for the Zr, Rh and Pd catalysed hydrodefluorination of fluoroarenes.^52^

**Scheme 12:**
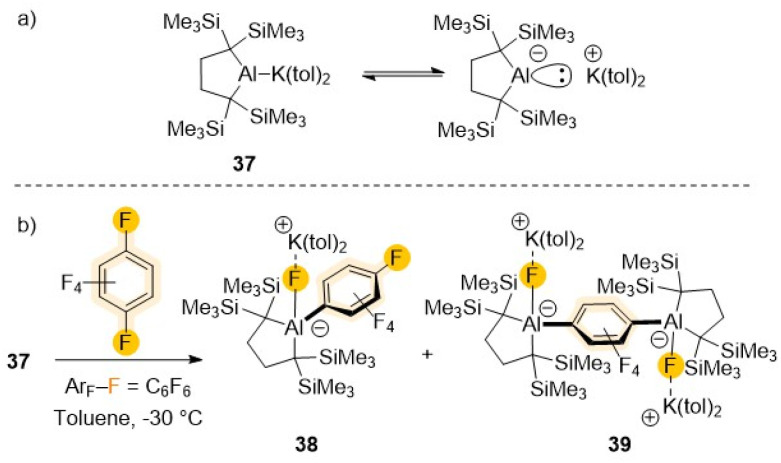
(a) Six-electron dialkylaluminium anion 37; (b) reactivity of 37 towards C_6_F_6_.

Recent advances on the synthesis and reactivity of aluminium(i) anions have revealed their strong nucleophilic and basic character equips them to activate a variety of small molecules including H_2_, ketones, P_4_ or CO_2_ to name but a few.^13*a*,*b*^ They have also uncovered unique reactivities towards sp^2^ C–H bond activation of non-activated arenes such benzene, toluene or naphthalene.^14,53,54^ Recently Yamashita has isolated a potassium salt of non-stabilised dialkylaluminium anion containing an unprecedented two-centre-two-electron Al–K bond (37 in Scheme 12).^54^ UV-vis analysis and DFT studies confirmed the Al centre possesses a lone pair of electrons and an unoccupied p orbital which justifies its strong basicity (*e.g.*, room temperature deprotonation of benzene) as well as its nucleophilicity. Thus, this aluminyl potassium reacts readily with C_6_F_6_ already at −35 °C affording a mixture of mono- (38) and di-aluminated (39) complexes as products of one and two S_N_Ar substitution reactions respectively. While 39 could be isolated as the major product when 37 was treated with 0.5 equiv. of C_6_F_6_, attempts to isolate 38 using a large excess of the fluoroarene still led to the formation of mixtures of 38 and 39. These findings are consistent with monosubstituted 38 being more reactive towards 37 than C_6_F_6_.^54*a*^ A plausible rationale for this reactivity pattern could be that the (dialkyl)(fluoro)alumanidyl substituent present in 38 causes a decrease in the electron density of the C_6_F_5_ fragment in comparison with that of C_6_F_6_, favouring a second S_N_Ar step, leading to formation of the di-aluminated product 39. The same group has also reported the reactivity of 37 towards fluorobenzene.^54*b*^ In this case no metalated intermediates could be isolated, however when the reaction was carried out at room temperature for 5 hours followed by iodine interception furnished a mixture of 3-fluoroiodobenzene (25%) and iodobenzene (28%). While the formation of iodobenzene is most likely *via* a S_N_Ar substitution at the *ipso* carbon (as found for the reaction of C_6_F_6_, Scheme 12), the presence of 3-fluoroiodobenzene is most likely due to the initial formation of a *meta*-fluorophenyl-substituted aluminate resulting from a C–H activation process.^54*b*^

**Scheme 13:**
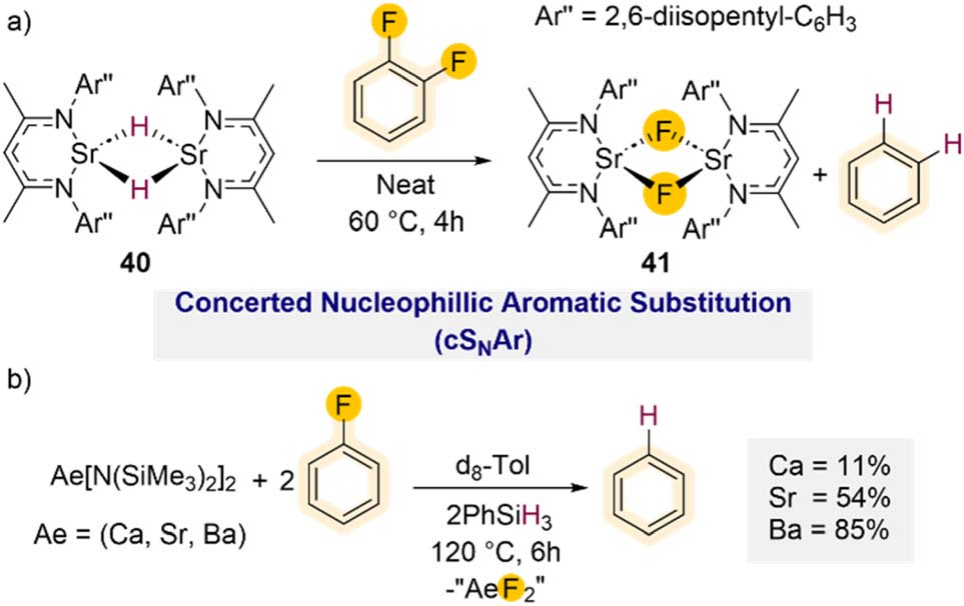
(a) Hydrodefluorination of 1,2-difluorobenzene using a super bulky powerful heavy alkaline earth (Ae) metal hydride complex 40; (b) hydrodefluorination of fluorobenzene using *in situ* generated hydride clusters *via* a combination of Ae[(N(SiMe_3_)_2_)]_2_.

Diverging from these C–F bond activation processes where the C–F bond cleavage of the takes place through oxidative addition, studies by Harder have demonstrated that powerful heavy alkaline earth (Ae) metal hydride complexes (Ca, Sr, Ba) can efficiently promote hydrodefluorination of fluoroarenes, including fluorobenzene *via* direct nucleophilic substitution.^55^ Using a well-defined strontium hydride complex stabilised by a super bulky β-diketiminate ligand [(^Dipep^Nacnac)SrH]_2_ (40) (^Dipep^Nacnac = Ar′′NC(Me)CHC(Me)NAr′′; Ar′′ = 2,6-diisopentyl–C_6_H_3_) smooth hydrodefluorination of 1,2-difluorobenzene could be achieved forming benzene and the subsequent Ca–F complex [(^Dipep^Nacnac)SrF]_2_ (41) (Scheme 13a) after 4 h at 60 °C.

Using fluorobenzene required significantly harsher reaction conditions (60 °C for 3 days) which led to the formation of ill-defined products. DFT calculations suggest that these C–F bond activations take place *via* a concerted nucleophilic aromatic substitution mechanism (cS_N_Ar). This is in agreement with a previous mechanistic proposal reported by Hill and Maron for hydrodehalogenation of ArX (X = Cl, Br) using Ca hydride complex [(^Dipp^Nacnac)CaH]_2_.^51*b*^ Furthermore, the efficient hydrodefluorination of 2,6-dimethyl-fluorobenzene offers further support to this proposed cS_N_Ar reaction path, excluding a possible aryne type mechanism as the *ortho*-positions are blocked for this substrate.

The Harder group has also shown that Group 2 metal amide complexes Ae[N(SiMe_3_)_2_]_2_ (Ae = Ca, Sr, Ba) react *in situ* with PhSiH_3_ to form large hydride clusters.^56^ While these systems are less well defined, they are remarkably robust and can tolerate high temperatures. Interestingly when assessing the reactivity of Ae[N(SiMe_3_)_2_]_2_/PhSiH_3_ mixtures towards fluorobenzene it was found that while the Ba amide can promote the hydrodefluorination reaction after 6 h at 120 °C (85% conversion), the lighter Sr and Ca amides provided reduced conversions of 54% and 11% respectively under the same conditions (Scheme 13b), evidencing a clear correlation between the metal size and their conversion rates.^55^

Related to this work, Okuda and Maron have also reported the ability of dicationic dimeric Ca hydride complex [(Me_4_TACD)_2_Ca_2_(μ-H)_2_(THF)][BAr_4_]_2_ (42) (Me_4_TACD = 1,4,7,10-tetramethyl-1,4,7,10-tetraazacyclododecane; BAr_4_ = B(C_6_H_3_-3,5-Me_2_)_4_) to promote the C–F bond cleavage of fluorobenzene furnishing fluoride complex [(Me_4_TACD)_2_Ca_2_(μ-F)_2_(THF)][BAr_4_]_2_ (43) along with benzene.^57^ Forcing reaction conditions are required (two days at 60 °C) to promote this hydrodefluorination process which is proposed to occur *via* a nucleophilic aromatic substitution mechanism.

Predating these studies, Crimmin has also showed that [^Dipp^NacnacMg(alkyl)] species were found to react with fluoroarenes under forcing conditions (80 °C, 1–168 h, excess of substrate) *via* a similar S_N_Ar type mechanism. The reactive Mg-alkyl species is actually formed *in situ via* C–F activation of a fluoroalkane by Mg(i) species 16. Once formed the Mg-alkyl species can deliver its alkyl substituent to the fluoroarene leading to the intermolecular C–C bond formation by heterocoupling of two C–F bonds.^58^

## Conclusions & outlook

Main group metal-mediated strategies have emerged as a more sustainable and regioselective alternative to precious transition-metals for functionalisation of C–H and C–F bonds of fluorinated aromatic molecules. Underpinning this research is two main approaches; the use of bespoke s-block containing bimetallic complexes where the cooperation of two metals is vital to the unique reactivity observed or the use of specially designed ligands to support main group metals where even in some cases unusually low oxidation states can be tolerated. The aforementioned bimetallic complexes overcome the limitations of classical deprotonative metalation strategies of fluoroarenes in forming more stable metal–carbon bonds in these delicate fluorinated anions avoiding unwanted side reactions and the use of cryogenic temperatures. On the other hand, the use of sterically demanding ligand sets for stabilisation of main group metals in low valent oxidation states allows the more challenging C–F bond functionalisation of these substrates mimicking the redox capabilities often observed with transition metal complexes and not typically attributed to main group metal complexes. Along with these protocols, the rational design of highly nucleophilic Group 2 metal hydride complexes has also opened new ground towards hydrodefluorination of non-activated fluoroarenes *via* nucleophilic aromatic substitution.

The selective functionalisation of fluoroarenes is an imperative methodology in the synthetic chemist's toolbox and the novel main group metal strategies described in this Perspective article provide complementary alternatives to the established methods used in research laboratory's today. Some of these main-group-metal-mediated approaches show excellent regio- and chemoselectivities while offering high functional group tolerances. This can have a profound effect on the design of future applications for late-stage functionalisation of organic molecules containing fluoroarene substituents. Furthermore, while this is an evolving field, preliminary studies have also shown the ability of the resulting fluoroaryl fragments present in these metal complexes (obtained either *via* C–H deprotonation or C–F bond activation) to engage in C–C bond forming processes such as Negishi cross-couplings. These findings greatly expand the synthetic potential of these approaches. Considering the beneficial role of fluorine in pharmaceuticals, agrochemicals and materials, the development of novel and sustainable approaches using earth abundant and non-toxic metals for the efficient functionalisation of partially fluorinated building blocks that can then be incorporated into more complex molecular scaffolds is particularly appealing.

Looking forward, judging by the recent and significant advances showcased in this Perspective, it is anticipated that alternative main group metal approaches for the C–H/C–F functionalisation of fluoroarenes will continue to develop at a steady pace. Many challenges still remain in this area, which include the use of less forcing conditions for the C–F bond activation of non-activated substrates, the extension of current studies to more complex organic substrates, as well as the implementation of some of the stoichiometric protocols to catalytic regimes.

## Author contributions

N. R. J., A. L. and E. H. conceptualized, wrote, and edited the Perspective.

## Conflicts of interest

There are no conflicts of interest to declare.

## Supplementary Material
